# Effect of Fiber and Fecal Microbiota Transplantation Donor on Recipient Mice Gut Microbiota

**DOI:** 10.3389/fmicb.2021.757372

**Published:** 2021-10-13

**Authors:** Yifan Zhong, Jiahong Cao, Zhaoxi Deng, Yanfei Ma, Jianxin Liu, Haifeng Wang

**Affiliations:** The Key Laboratory of Molecular Animal Nutrition, Ministry of Education, College of Animal Science, Zhejiang University, Hangzhou, China

**Keywords:** fecal microbiota transplantation, diet fiber, wild pig, domestic pig, gut microbiota

## Abstract

Both fecal microbiota transplantation (FMT) and dietary fiber intervention were verified as effective ways to manipulate the gut microbiota, whereas little is known about the influence of the combined methods on gut microbiota. Here, we constructed “non-industrialized” and “industrialized” gut microbiota models to investigate the donor effect of FMT and diet effect in shaping the gut microbiota. Mice were transplanted fecal microbiota from domestic pig and received a diet with low-fiber (D) or high-fiber (DF), whereas the other two groups were transplanted fecal microbiota from wild pig and then received a diet with low-fiber (W) or high-fiber (WF), respectively. Gut microbiota of WF mice showed a lower Shannon and Simpson index (*P* < 0.05), whereas gut microbiota of W mice showed no significant difference than that of D and DF mice. Random forest models revealed the major differential bacteria genera between four groups, including *Anaeroplasma* or unclassified_o_*Desulfovibrionales*, which were influenced by FMT or diet intervention, respectively. Besides, we found a lower out-of-bag rate in the random forest model constructed for dietary fiber (0.086) than that for FMT (0.114). Linear discriminant analysis effective size demonstrated that FMT combined with dietary fiber altered specific gut microbiota, including *Alistipes*, *Clostridium* XIVa, *Clostridium* XI, and *Akkermansia*, in D, DF, W, and WF mice, respectively. Our results revealed that FMT from different donors coupled with dietary fiber intervention could lead to different patterns of gut microbiota composition, and dietary fiber might play a more critical role in shaping gut microbiota than FMT donor. Strategies based on dietary fiber can influence the effectiveness of FMT in the recipient.

## Introduction

Gut microbiota contains highly diverse microorganisms, which have metabolic, immune, and protective functions and play a vital role in gut homeostasis, and host health (Goldsmith and Sartor, 2014). Fecal microbiota transplantation (FMT) is used to restore the healthy status of the dysbiosis microbiome *via* introducing the healthy microbiota to the gut. Functioning similarly to probiotics, FMT helps to maintain the microbiotal balance and function. From a microbiological perspective, FMT success can also be defined by a shift in the gut microbiome profile of an individual toward that of the donor (Wilson et al., 2019). Given the implementation of FMT programs, donor selection is a fundamental challenge (Bibbò et al., 2020) and is a crucial component in FMT success (Moayyedi et al., 2015; Vermeire et al., 2016).

Numerous studies have revealed that diet plays an important role in mediating the composition and metabolic function of gut microbiota (Sonnenburg and Bäckhed, 2016). Consumption of dietary fiber interacts directly with gut microbes and leads to the production of key metabolites such as short-chain fatty acids (SCFAs), thus impacting gut microbial ecology, host physiology, and health (Makki et al., 2018; Chang et al., 2021). An industrialized diet consisting of highly refined foods and low in dietary fiber usually induces the dysfunctional intestinal barrier and chronic diseases (Martínez et al., 2015; Yeagle, 2015; García-Montero et al., 2021). When industrialized and non-industrialized populations were compared, the significant differences within the gut microbiota composition was observed, suggesting that bacterial can be driven by differences in diet, including dietary fiber (De Filippo et al., 2010). Mechanistic studies and clinical trials on isolated and extracted fibers have demonstrated promising regulatory effects on the gut microbiota (Gill et al., 2021).

Pig is one of the earliest domesticated livestock species and received an “industrialized” diet in modern feedlots. Thus, supplementation of dietary fiber can be a beneficial modulation of the gut microbiome and has high impactions on humans and livestock that sustain societal needs (Slavin, 2013; Michalak et al., 2020). Previous studies have implicated the supplementation of dietary as a potential feeding strategy to control gut dysfunction by decreasing the retention time of digesta and reducing the proliferation of pathogens in the gut of domestic pigs (Kim et al., 2012; Heo et al., 2013). Unlike other domesticated animals, wild ancestors of domesticated pigs still exist in large numbers in the wild environment (Vigne, 2011). Wild pig mainly feeds on acorns, wild fruits, grassroots, and stems, so the diet of wild boar may have higher cellulose content and lower carbohydrate or fat content (Rivero et al., 2019). When the gut microbiota of specific-pathogen-free pigs, domestic pigs, and wild pigs was compared, specific operational taxonomic units (OTUs) enriched in the gut of wild pigs might contribute to higher resistance to African swine fever than those susceptible animals (Correa-Fiz et al., 2019).

The study of pigs has excellent potential to inform humans due to the many similarities identified in the physiological attributes of these two species. It has been shown that humans share more similarities with pigs in anatomy, genetics, physiology, pharmaceutical bioavailability, and nutrient digestibility than with rodents (Wang et al., 2016). Hence, the pig is a superior model to rodents for studying human physiology and pathology on enteric health. In this study, FMT was used to establish mouse models of gut microbiota in wild and domestic pigs after indigenous microbiota was depleted by antibiotics. The mice were provided with a high-fiber diet and a low-fiber diet to evaluate the effect of FMT and dietary fiber intervention on the gut microbiota, which will help better understand the utilization of both FMT and diet intervention on gut microbiota.

## Materials and Methods

### Animals

All procedures involving animals are fully implemented in accordance with the “Regulations on the Use of Laboratory Animals” of Zhejiang Province, China. This research was specially approved by the Animal Protection and Use Committee of Zhejiang University (ethics license ZJU20170529). Male ICR mice (body weight 20 ± 2 g, 8 weeks old) were obtained from the Model Animal Research Center of Nanjing University (Nanjing, China). Mice were kept at a temperature of 25°C with 12-h light–dark cycle and provided food and water at will.

### Experimental Design

Forty male ICR mice were randomly divided into four groups (10 mice/group): transplant wild pig fecal microbiota and feed low-fiber diet (W), transplant wild pig fecal microbiota and feed a high-fiber diet (WF), transplant domestic pig fecal microbiota and feed a low-fiber diet (D), and transplant domestic pig fecal microbiota and feed a high-fiber diet (DF). High- and low-fiber (cellulose and inulin) diet intervention lasted for 28 days. The composition of customized commercial chow diet (Slacom), low-fiber diet, and high-fiber diet is shown in Supplementary Table 1.

### Depletion of the Gut Microbiota

To remove the commensal gut microbiota, ICR mice were transferred to sterile cages and treated by adding ampicillin (1 g/L; Ratiopharm), neomycin sulfate (1 g/L; Sigma), vancomycin (500 mg/L; Cell Pharm), and Mtz (1 g/L; Fresenius) to the drinking water casually for 2 weeks.

### Fecal Microbiota Transplantation

Fresh fecal pellets were collected from eight wild pigs and eight domestic pigs; the composition of gut microbiota in wild pigs and domestic pigs are shown in Supplementary Figure 1. For FMT, fecal pellets were diluted with sterile phosphate-buffered saline (1 g/ml). In short, the feces were soaked in sterile phosphate-buffered saline for approximately 15 min and then centrifuged at 1,000 rpm, 4°C for 5 min to obtain total microbiota. The final bacterial suspension was mixed with an equal volume of 40% sterile glycerol to a final concentration of 20% (w/v) and then stored at −80°C until transplantation. For the transplantation, 200 μl of bacterial suspension was immediately transplanted into each recipient mouse every day for 14 consecutive days.

### 16S Ribosomal RNA Gene Sequencing

After a dietary intervention, samples of mouse colon contents were collected in sterile microtubes, immediately frozen in liquid nitrogen, and stored in a refrigerator at −80°C until analysis. Microbial DNA was extracted from colon contents using the DNA extraction kit according to the manufacturer’s protocols. The V3-V4 region of the microbiota 16S ribosomal RNA genes was amplified by polymerase chain reaction (95°C for 3 min, followed by 30 cycles at 98°C for 20 s, 58°C for 15 s, and 72°C for 20 s and a final extension at 72°C for 5 min) using primers 341F 5′-CCTACGGGRSGCAGCAG)-3′ and 806R5′-GGACTACVVGGGTATCTAATC-3′. The polymerase chain reaction reactions were performed in a 30-μl mixture containing 15 μl of 2 × KAPA Library Amplification Ready Mix, 1 μl of each primer (10 μM), 50 ng of template DNA, and double-distilled water. Amplicons were extracted from 2% agarose gels and purified using the AxyPrep DNA Gel Extraction Kit (Axygen Biosciences, Union City, CA, United States) according to the manufacturer’s instructions and quantified using Qubit^®^2.0 (Invitrogen, United States). After preparation of the library, these tags were sequenced on the HiSeq platform (Illumina, Inc., CA, United States) for paired-end reads of 250 bp, which were overlapped on their ends for concatenation into original longer tags. DNA extraction, library construction, and sequencing were conducted at Realbio Genomics Institute (Shanghai, China). Tags, trimmed of barcodes and primers, were further checked on their rest lengths and average base quality. 16S tags were restricted between 220 and 500 bp such that the average Phred score of bases was no worse than 20 (Q20) and no more than 3 ambiguous N. The copy number of tags was enumerated, and redundancy of repeated tags were removed. Only the tags with a frequency of more than 1, which tend to be more reliable, were clustered into operational taxonomic units (OTUs), each of which had a representative tag. OTUs were clustered with 97% similarity using UPARSE,^1^ and chimeric sequences were identified and removed using Userach (version 7.0). Each representative tag was assigned to taxa by RDP Classifier^2^ against the RDP database (see text footnote 2) using a confidence threshold of 0.8. OTU profiling table and alpha/beta diversity analyses were also achieved by python scripts of QIIME1 (v1.9.1). All DNA sequences were deposited in the National Center for Biotechnology Information sequence read archive with the project number PRJNA723114.

### Statistical Analysis

The random forest model was applied to classify different treatments, and the accuracy was evaluated with an out-of-bag (OOB) value. A score reflecting the importance with mean decrease accuracy (MDA) in the model was given to each genus based on the increase in error caused by removing that microbiota from the predictors. Linear discriminant analysis effect size (LEfSe) was used to screen differential microbiota between four groups. The abundances of microbiota among the four groups were compared using the Kruskal–Wallis *H*-test, and a non-parametric Scheirer–Ray–Hare test was applied for the evaluation of donor, fiber, and interaction effect. The *P* < 0.05 was considered to indicate statistical significance. The analysis of the datasets was completed with R software (version 3.5.1).

## Results

### 16S Ribosomal RNA Gene Sequencing Data of Gut Microbiota

**FIGURE 1:**
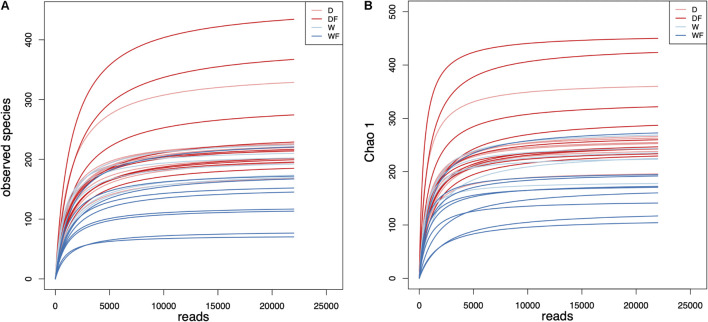
Rarefaction curves of **(A)** observed species and **(B)** Chao 1 index for gut microbiota diversity of mice transplanted domestic pig fecal bacteria and feed a low-fiber diet (D) or high-fiber diet (DF), transplanted wild boar fecal bacteria and feed low-fiber diet (W) or high-fiber diet (WF).

Intestinal contents of the mice were used for amplicon sequencing, and a total of 1,225,209 high-quality reads were achieved. The average numbers of high-quality reads generated from gut microbiota were 35,005, and sequence lengths were concentrated in 400–420 (Supplementary Table 2). Based on 97% sequence similarity, all the sequences were clustered into 797 bacterial OTUs. As shown in Figure 1, the rarefaction curve of observed species and Chao 1 index of gut microbiota plateaued with the increase of reads.

### Diversity of Gut Microbiota

**FIGURE 2:**
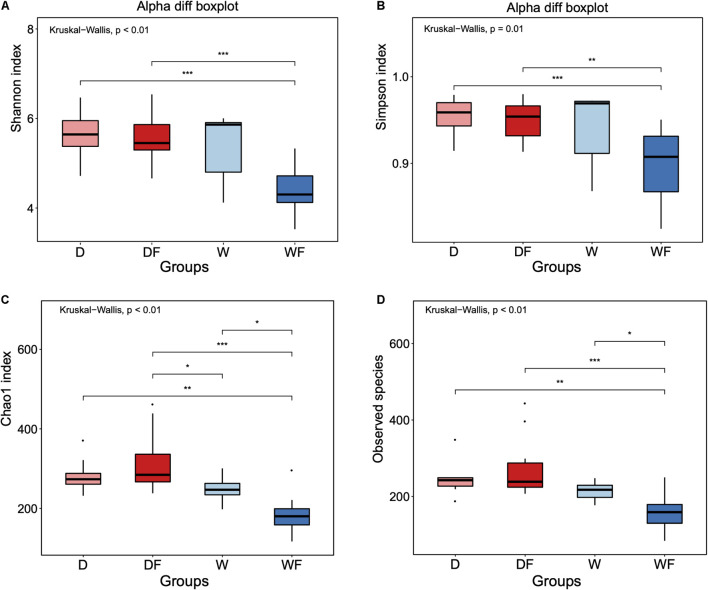
Alpha diversity of **(A)** Shannon index, **(B)** Simpson index, **(C)** Chao 1 index, and **(D)** Observed species for gut microbiota diversity of mice transplanted domestic pig fecal bacteria and feed a low-fiber diet (D) or high-fiber diet (DF), transplanted wild boar fecal bacteria and feed low-fiber diet (W) or high-fiber diet (WF). *p < 0.05, **p < 0.01, and ***p < 0.001.

The alpha diversity of gut microbiota between four groups was evaluated (Figure 2). Significant differences (*P* < 0.05) of Shannon index (Figure 2A), Simpson index (Figure 2B), Chao 1 index (Figure 2C), and observed species (Figure 2D) between four groups were observed. There was no significant difference between the mice FMT from the domestic pig (D) and received with high dietary fiber (DF) and mice FMT from wild pig (W) groups in Shannon index, Simpson index, and observed species. The mice FMT from wild pig and received with high dietary fiber (WF) showed the lowest diversity index within four groups, including Shannon, Simpson, Chao 1, and observed species.

**FIGURE 3:**
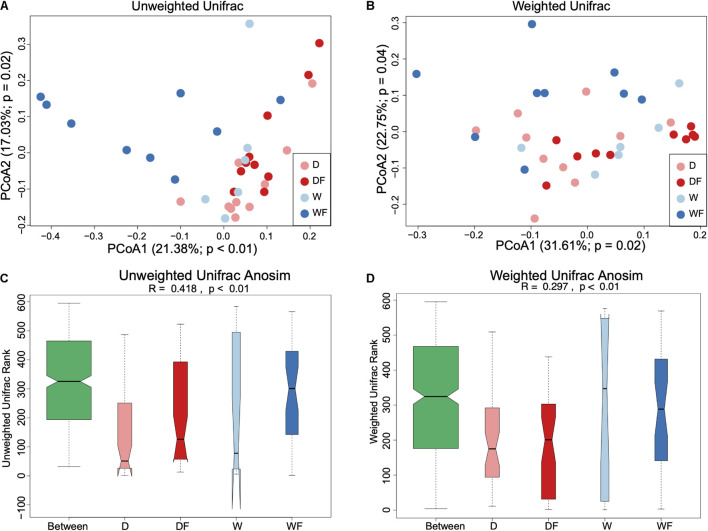
Beta diversity of gut microbiota between mice transplanted domestic pig fecal bacteria and feed a low-fiber diet (D) or high-fiber diet (DF), transplanted wild boar fecal bacteria and feed low-fiber diet (W) or high-fiber diet (WF). Principal coordinates analysis with unweighted Unifrac distance **(A)**, weighted Unifrac distance **(B)**, and Anosim analysis for unweighted Unifrac **(C)** and weighted Unifrac **(D)** applied for gut microbiota between D, DF, W, and WF groups.

Principal coordinate analysis based on weighted UniFrac and unweighted UniFrac distance metrics together with analysis of similarities (Anosim) was performed (Figure 3). Both principal coordinate analysis plots of unweighted (Figure 3A) and weighted (Figure 3B) Unifrac distance showed a significant difference in the structure of the gut microbiota (*P* < 0.05). Anosim also showed a significant difference between the four groups, both in unweighted (Figure 3C) and weighted (Figure 3D) Unifrac distance.

### Composition of Gut Microbiota

**FIGURE 4:**
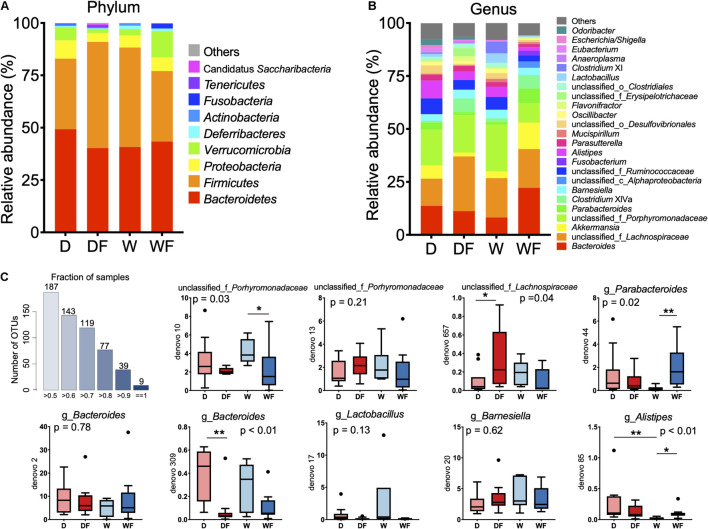
Relative abundance of gut microbiota in mice transplanted domestic pig fecal bacteria and feed a low-fiber diet (D) or high-fiber diet (DF), transplanted wild boar fecal bacteria and feed low-fiber diet (W) or high-fiber diet (WF). **(A)** Gut microbiota in mice of four groups at phylum level with relative abundance over 0.1% in at least one group was shown. **(B)** Gut microbiota in mice of four groups at genus level with relative abundance over 1% in at least one group was shown. **(C)** Boxplot of relative abundance of nine OTUs existed in gut of all individuals. *p < 0.05, **p < 0.01.

The relative abundance (Table 1) and composition (Figure 4) of gut microbiota between four groups were shown. At the phylum level (Figure 4A), the gut microbiota was dominated by *Bacteroidetes* and *Firmicutes*, which accounted for 77–91% within the four groups. The relative abundance of *Firmicutes*, *Verrucomicrobia*, *Deferribacteres*, *Tenericutes*, and Candidatus *Saccharibacteria* showed a significant difference (*P* < 0.05) between the four groups (Table 1). The DF group showed the highest level of relative abundance of *Firmicutes*, *Tenericutes*, and unclassified_k_Bacteria and the lowest level of relative abundance of *Verrucomicrobia* and *Deferribacteres* (*P* < 0.05) in the gut than other groups.

**TABLE 1:** Relative abundance of differential gut microbiota at genus level (> 0.1%) between mice transplanted domestic pig fecal bacteria and feed a low-fiber diet (D) or high-fiber diet (DF), transplanted wild boar fecal bacteria and feed low-fiber diet (W) or high-fiber diet (WF).

	D	DF	W	WF	SEM	Donor	Fiber	Interaction
*Bacteroidetes*	49.26	40.33	40.81	43.46	2.05	0.76	0.32	0.12
*Bacteroides*	13.73	11.23	8.24	22.21	1.79	0.39	0.39	0.02
*Barnesiella*	3.10	4.04	4.03	3.34	0.35	0.86	0.65	0.24
*Butyricimonas*	0.58^a^	0.09^c^	0.24^b^	0.06^c^	0.05	0.31	<0.01	0.18
*Odoribacter*	2.76^a^	1.38^a^	0.00^b^	0.00^b^	0.42	<0.01	0.77	0.89
*Parabacteroides*	3.36^a^	1.35^b^	1.19^b^	6.84^a^	0.64	0.29	0.89	<0.01
unclassified_f_*Porphyromonadaceae*	16.76^a^	17.67^a^	22.15^a^	9.11^b^	1.40	0.19	0.11	0.03
*Prevotella*	0.03	0.22	0.11	0.05	0.05	0.29	0.91	<0.01
*Alistipes*	8.37^a^	4.04^b^	4.81^b^	1.85^c^	0.60	<0.01	<0.01	0.83
unclassified_o_*Bacteroidales*	0.45^a^	0.13^b^	0.00^c^	0.00^c^	0.04	<0.01	0.03	0.08
*Firmicutes*	33.72^b^	50.65^a^	47.46^ab^	33.58^b^	2.60	0.53	0.42	<0.01
*Lactobacillus*	1.37^a^	0.38^b^	4.43^a^	0.18^b^	0.55	0.65	<0.01	0.40
*Lactococcus*	0.08^a^	0.02^b^	0.13^a^	0.02^b^	0.01	0.54	<0.01	0.52
*Streptococcus*	0.18^a^	0.65^a^	0.25^a^	0.05^b^	0.15	0.17	<0.01	0.21
*Clostridium sensu stricto*	0.01^a^	0.01^a^	0.90^a^	0.00^b^	0.11	0.25	0.02	<0.01
*Eubacterium*	1.72^a^	0.19^bc^	0.51^b^	0.08^c^	0.18	<0.05	<0.01	0.83
*Anaerostipes*	0.00^b^	0.00^b^	0.00^b^	0.51^a^	0.08	0.04	0.03	0.40
*Blautia*	0.02^ab^	0.01^ab^	0.00^bc^	0.31^a^	0.05	0.27	0.14	<0.01
*Clostridium* XlVa	0.81^bc^	6.55^a^	1.74^ab^	6.52^a^	1.03	0.94	<0.01	0.19
*Clostridium* XlVb	0.62^a^	0.61^a^	0.62^a^	0.19^b^	0.11	0.36	0.01	0.29
*Coprococcus*	0.11	0.02	0.00	0.00	0.03	0.16	0.65	0.44
*Lachnospiracea incertae sedis*	0.20^ab^	0.06^b^	0.78^a^	0.01^b^	0.06	0.43	<0.01	0.15
*Roseburia*	0.78	0.11	0.68	0.91	0.22	0.62	0.14	0.21
unclassified_f_*Lachnospiraceae*	12.83	25.83	18.59	18.36	2.08	0.69	0.11	0.04
*Clostridium* XI	0.64^b^	0.04^c^	5.56^ab^	0.16^ac^	0.88	0.68	<0.01	0.21
*Anaerotruncus*	0.36^a^	0.17^ab^	0.37^a^	0.13^b^	0.03	0.43	<0.01	0.53
*Clostridium* IV	0.41^a^	0.13^b^	0.45^ab^	0.11^ab^	0.04	0.94	<0.01	0.83
*Faecalibacterium*	0.27	0.16	0.02	0.03	0.07	0.53	0.20	0.19
*Flavonifractor*	0.92	2.17	0.68	0.47	0.26	0.03	0.92	0.22
*Gemmiger*	0.05	0.11	0.01	0.01	0.02	0.44	0.16	0.28
*Oscillibacter*	1.47^a^	1.40^a^	1.98^a^	0.64^b^	0.16	0.26	0.04	0.08
unclassified_f_*Ruminococcaceae*	7.36^a^	4.60^a^	6.04^a^	2.84^b^	0.55	0.14	<0.01	0.84
unclassified_o_*Clostridiales*	1.58^a^	1.88^a^	2.02^a^	0.20^b^	0.24	0.01	0.06	<0.01
unclassified_c_*Clostridia*	0.18	0.22	0.13	0.12	0.02	0.08	0.96	0.74
*Allobaculum*	0.31	0.05	0.37	0.17	0.07	0.63	0.63	0.23
*Clostridium* XVIII	0.34^a^	0.01^b^	0.12^a^	0.34^a^	0.06	0.39	0.03	0.03
*Erysipelotrichaceae incertae sedis*	0.10	0.01	0.05	0.08	0.02	0.09	0.11	0.33
*Turicibacter*	0.03^ab^	0.03^a^	0.17^ab^	0.00^b^	0.02	0.06	0.33	0.03
unclassified_f_*Erysipelotrichaceae*	0.13^b^	3.86^a^	0.26^a^	0.28^a^	0.46	0.09	0.08	0.06
*Megamonas*	0.25	0.07	0.01	0.01	0.07	0.20	0.81	0.57
*Veillonella*	0.01	0.12	0.00	0.01	0.02	0.07	0.16	0.72
unclassified_p_*Firmicutes*	0.31	0.68	0.45	0.78	0.13	0.70	0.39	0.11
*Proteobacteria*	8.75	4.27	5.75	6.56	0.71	0.71	0.05	0.06
unclassified_c_*Alphaproteobacteria*	0.05^b^	0.03^b^	0.05^b^	3.00^a^	0.42	0.02	0.27	<0.01
*Parasutterella*	2.79	2.57	2.13	1.38	0.53	0.15	0.30	0.63
*Sutterella*	0.01^b^	0.02^b^	0.00^b^	0.84^a^	0.12	0.10	0.02	0.08
*Bilophila*	0.19	0.06	0.12	0.15	0.02	0.19	0.27	0.04
unclassified_o_*Desulfovibrionales*	3.89^a^	0.65^c^	2.67^ab^	0.95^bc^	0.34	0.74	<0.01	0.12
*Escherichia/Shigella*	1.52	0.34	0.17	0.03	0.32	0.07	0.65	0.41
*Klebsiella*	0.03	0.13	0.04	0.03	0.03	0.34	0.57	0.14
*Acinetobacter*	0.02	0.06	0.11	0.02	0.02	0.07	0.93	0.09
*Pseudomonas*	0.05	0.10	0.16	0.02	0.03	0.09	0.70	0.10
*Verrucomicrobia*	6.34^b^	1.84^c^	3.12^bc^	12.49^a^	1.04	<0.01	0.60	<0.01
*Akkermansia*	6.34^b^	1.84^c^	3.12^b^	12.49^a^	1.04	<0.01	0.60	<0.01
*Actinobacteria*	0.88	0.48	1.11	0.41	0.13	0.65	0.07	0.87
*Bifidobacterium*	0.50	0.26	0.41	0.31	0.08	0.77	0.78	0.57
*Enterorhabdus*	0.17^a^	0.07^b^	0.29^a^	0.02^c^	0.03	0.25	<0.01	0.14
*Olsenella*	0.18^a^	0.02^bc^	0.30^a^	0.05^ab^	0.04	0.51	<0.01	0.25
*Deferribacteres*	0.44^ab^	0.34^bc^	1.57^a^	1.00^a^	0.18	0.01	0.69	0.21
*Mucispirillum*	0.44^ab^	0.34^bc^	1.57^a^	1.00^a^	0.18	0.01	0.69	0.21
*Tenericutes*	0.27^b^	1.06^a^	0.04^c^	0.14^c^	0.13	<0.01	0.39	0.29
*Anaeroplasma*	0.27^b^	1.05^a^	0.04^c^	0.14^c^	0.13	<0.01	0.44	0.34
unclassified_k_Bacteria	0.16^b^	0.42^a^	0.03^c^	0.13^b^	0.03	<0.01	<0.01	0.74
unclassified_k_Bacteria	0.16^b^	0.42^a^	0.03^c^	0.13^b^	0.03	<0.01	0.02	0.74
*Candidatus Saccharibacteria*	0.01^b^	0.46^a^	0.03^b^	0.02^b^	0.05	<0.01	<0.01	0.02
*Saccharibacteria genera incertae sedis*	0.01^b^	0.46^a^	0.03^b^	0.02^b^	0.05	<0.01	<0.01	0.02
*Fusobacteria*	0.15	0.05	0.04	2.13	0.29	0.07	0.15	0.22
*Fusobacterium*	0.15	0.04	0.04	2.13	0.29	0.07	0.17	0.20
Others	0.61	1.22	0.63	0.34	0.19	0.07	0.06	0.50

*^a,b,c^Means with same row followed by different superscripts differ at P < 0.05.*

At the genus level, gut microbiota composition (relative abundance > 1%) is shown in Figure 4B, and differential microbiota genus (relative abundance > 0.1%) is shown in Table 1. The differential bacterial genera in the gut among the four groups were mainly from the phylum *Bacteroidetes*, *Firmicutes*, and *Proteobacteria*. The genus *Odoribacter* and unclassified_o_*Bacteroidales* from *Bacteroidetes* were not detected in the W and WF groups. The relative abundance of *Butyricimonas* in the gut of DF and WF mice was significantly lower than that in D and W mice. The lowest relative abundance of unclassified_f_*Porphyromonadaceae* was observed in the gut of WF mice, whereas no significant difference was observed between D, DF, and W mice. A total of 19 microbiota genera from the phylum *Firmicutes* showed a significant difference among the four groups. Within the 19 differential genera, *Clostridium sensu stricto* and *Turicibacter* were not detected, whereas *Anaerostipes* was unique in the gut of WF mice. A lower abundance of *Lactobacillus* and *Lactococcus* was observed in DF and WF mice than in D and W mice. Lower abundance of *Clostridium* XIVb, *Oscillibacter*, unclassified_f_*Ruminococcaceae*, and unclassified_o_*Clostridiales* in the gut of WF mice were observed than in D, DF, and W mice. Relative abundance of the genus unclassified_c_*Alphaproteobacteria* and *Sutterella* from the phylum *Proteobacteria* and *Akkermansia* from the phylum *Verrucomicrobia* in the gut of WF mice was significantly higher than that in other groups. Besides, a lower abundance of *Enterorhabdus* from *Actinobacteria* and *Anaeroplasma* from *Tenericutes* in the gut of WF was observed. As shown in Figure 4C, nine OTUs were found to exist in all the samples, which belonged to the family *Porhyromonadaceae* and *Lachnospiraceae* or the genus *Branesiella*, *Parabacteroides*, *Lactobacillus*, *Bacteroides*, and *Alistipes*, respectively.

### Random Forest and Linear Discriminant Analysis Effect Size Analysis

**FIGURE 5:**
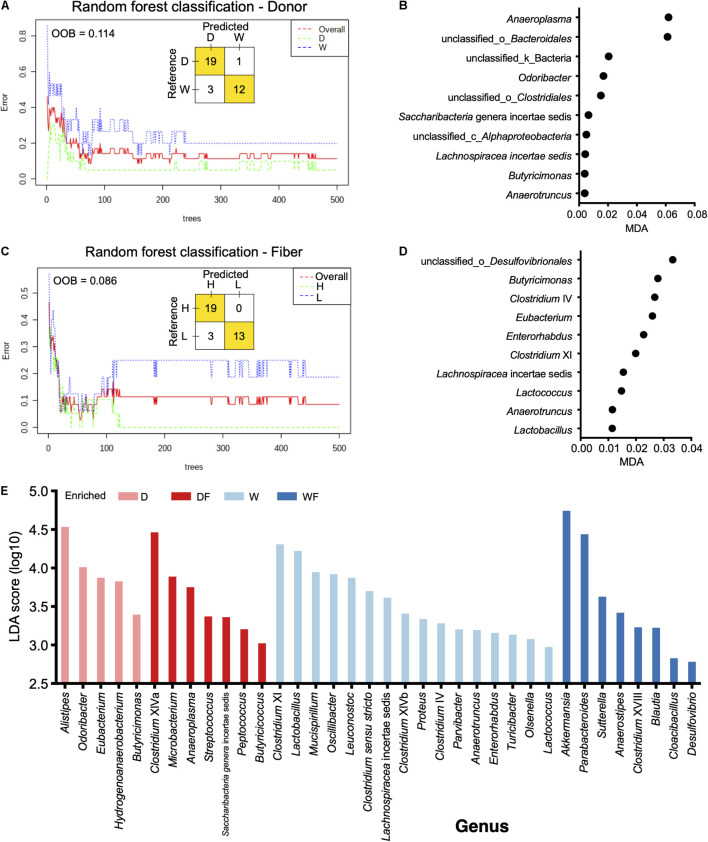
Random forest (RF) model and linear discriminant analysis effect size revealed differential genera between mice with different treatments. **(A)** RF model constructed for classification mice transplanted domestic pig (D) and wild boar (W) fecal bacteria. **(B)** Bacteria genera with top 10 mean decrease accuracy selected by RF model for classification of donor effect. **(C)** RF model constructed for classification mice treated with low-fiber (L) and high-fiber (H) diets. **(D)** Bacteria genera with top 10 mean decrease accuracy selected by RF model for classification of diet effect. **(E)** Bar plot of linear discriminant analysis score of bacteria genera selected by linear discriminant analysis effect size (linear discriminant analysis score > 2).

The random forest models were constructed to discriminate gut microbiota in mice affected by the donor or fiber. As shown in Figure 5A, a random forest model was constructed for the discrimination of the donor effect to the gut microbiota in mice; the OOB rate was 0.114. The MDA values of bacteria genera are shown in Supplementary Table 3, and genera with top 10 MDA values are shown in Figure 5B, including *Anaeroplasma*, unclassified_o_*Bacteroidales*, unclassified_k_Bacteria, *Odoribacter*, unclassfifed_o_*Clostridiales*, *Saccharibacteria* genera incertae sedis, unclassified_c_*Alphaproteobacteria*, *Lachnospiracea* incertae sedis, *Butyricimonas*, and *Anaerotruncus*. We also constructed the random forest model for the classification of fiber effect on the gut microbiota in mice. The OOB rate was 0.086 (Figure 5C), and the MDA values of bacteria genera are shown in Supplementary Table 4. Bacteria genera with top 10 MDA values are shown in Figure 5D, including unclassified_o_*Desulfovibrionales*, *Butyricimonas*, *Clostridium* IV, *Eubacterium*, *Enerorhabdus*, *Clostridium* XI, *Lachnospiracea* incertae sedis, *Lactococcus*, *Anaerotruncus*, and *Lactobacillus*. The LEfSe algorithm analysis was used for high-dimensional biomarker discovery among D, W, DF, and WF groups (Figure 5E). Bacteria genera enriched in the gut of WF with a linear discriminant analysis score over two were observed, including *Akkermansia*, *Parabacteroides*, *Sutterella*, *Anaerostipes*, *Clostridium* XVIII, *Blautia*, *Cloacibacillus*, and *Desulfovibrio*.

## Discussion

Mice were transplanted with domestic or wild pig fecal microbiota and supplied the diet with differential dietary fiber. After that, gut contents were sampled and sequenced for the detection of bacterial composition in mice. The rarefaction curve of alpha diversity revealed enough observations, and sampling depth has been made in all samples.

There was no significant difference in alpha diversity index in the gut microbiota between the DF and the D group, whereas the Chao 1 index and observed species of gut microbiota in WF mice were significantly lower than that of the W mice. Differential alpha diversity index of gut microbiota in mice transported from the same donor was observed, suggesting that the gut microbiota composition can be shaped by differential dietary fiber (Makki et al., 2018). However, although the diet could shape the gut microbiota patterns, the gut microbiota composition of mice transported from differential donors also showed differential responses to the same diet. For instance, no significant differences in alpha diversity were observed between D and W mice, whereas significant differences in alpha diversity of DF and WF mice were observed. Although FMT has been applied as an effective therapy for the improvement of host health (Kelly et al., 2014; Chinna Meyyappan et al., 2020), contrasting experimental results on FMT were usually observed and challenging to interpret (Vendrik et al., 2020). Our results showed that the diet could be one of the reasons influencing the effectiveness of FMT. Thus, implementing of microbiome-targeted diet might be an essential strategy for the subsequent FMT to sustain the bacterial patterns in the gut (Zhang et al., 2019). The results suggested that both the FMT and diet intervention could influence the gut microbiota composition in mice.

Although mice received treatments and showed differential patterns of gut microbiota, nine specific bacteria OTUs were still observed existing in all samples, including unclassified_f_*Porhyromonadaceae*, unclassified_f_*Lachnospiraceae*, *Barnesiella*, *Parabacteroides*, *Lactobacillus*, *Bacteroides*, and *Alistipes*. As *Bacteroidetes* and *Firmicutes* were the dominant phyla in the gut, seven of nine OTUs were from the *Bacteroidetes*, and the rest of the OTUs were from *Firmicutes*. These core gut microbiota OTUs were reported to be pivotal to the host homeostasis in healthy mice of other studies (Xiao et al., 2015), suggesting that the mice with different treatments in our study might still keep the homeostasis of gut microbiota (Wang et al., 2019).

On the other hand, random forest models were constructed to reveal the effect of donor or diet fiber on the bacteria genera. When a random forest model was constructed to classify the donor effect of FMT, *Anaeroplasma* showed the highest MDA value in the random forest model, suggesting that FMT of the different donors had a more significant effect on the *Anaeroplasma* than other genera in the gut. A higher abundance of *Anaeroplasma* in mice FMT from domestic pigs was observed than that in mice FMT from the wild pig. The higher relative abundance of *Anaeroplasma* has been reported to be positively corrected with the digestibility in pigs (Niu et al., 2015). Studies suggested that mammalian gut bacterial genomes adapted to environmental changes imposed by their mammalian host (Koskella and Brockhurst, 2014; Zhao et al., 2019). Although the wild pig belongs to the same species as domestic pigs, the wild pig is suited to the wild habitat, and natural genetic selection has been driven by selection pressures of challenging environments, whereas phenotypes in commercial systems such as growth/production orientated are not necessary (Rivero et al., 2019). Higher relative abundance of *Anaeroplasma* in mice FMT from domestic pig might be results of the domestic pig that adapted for the growth/production.

The bacteria unclassified_o_*Bacteroidales* and *Odoribacter* were found to exist in the mice FMT from the domestic pig, whereas not detected in mice FMT from wild pig. *Odoribacter*, belonging to the order *Bacteroidales*, is a common member both in the human and pig gut microbiota and is capable of butyrate production *via* lysine fermentation and succinate reduction (Lim et al., 2017; Rychlik, 2020). Lack of *Odoribacter* suggesting differential ways in supplementary of SCFAs might exist in the wild pig and the mice FMT from the wild pig. Moreover, *Akkermansia* with the highest linear discriminant analysis score was observed to be enriched in WF mice, which has been reported to convert dietary fiber into acetate, propionate, and butyrate in the gut (Chambers et al., 2018; Xu et al., 2020). Although the relative abundance of SCFA-producing genera *Odoribacter* was depleted in mice FMT from the wild pig, a higher relative abundance of *Akkermansia* coupled with high dietary fiber in WF mice might be an alternative way to supply the SCFAs. The FMT from different donors contributed to the differential of specific genera in the gut of mice, although the mice received dietary fiber at different content. In return, specific genera have been altered by the differential dietary fiber contents rather than FMT, including unclassified_o_*Desulfovibrionales*, *Butyricimonas*, *Clostridium* IV, and *Eubacterium*. Higher relative abundance of unclassified_o_*Desulfovibrionales* and *Butyricimonas* were observed in the gut of mice received with lower dietary fiber. Bacteria from the order *Desulfovibrionales* were usually pro-inflammatory (Rajilić-Stojanović and de Vos, 2014) through colonization, utilization, and degradation of the colonic mucin layer (Earley et al., 2015; Bishehsari et al., 2018). Consistent with previous studies, *Butyricimonas*, *Clostridium* XI, and *Eubacterium* were more abundant in the gut with a low-fiber diet in mice or adults (Hamilton et al., 2015; Lin et al., 2018; Tran et al., 2019). A lower OOB rate in the model constructed in fiber than that in the model donor was observed, suggesting that the intervention of dietary fiber might play a more critical role in shaping the gut microbiota of mice.

Besides the bacteria independent from FMT or dietary fiber, LEfSe analysis revealed the bacteria genera influenced by FMT together with dietary fiber in mice. Enrichment of *Akkermansia* was observed in the gut of mice FMT from wild pig and received with higher dietary fiber. *Akkermansia* is a mucin degrader that converts mucin to SCFAs that may mediate the immunoregulatory effects (Derrien et al., 2004) and has been reported to be an emerging probiotic that can be enhanced through dietary interventions (Zhou, 2017). *Parabacteroides* was also observed to be enriched in WF mice and prominently found in healthy individuals’ gut (Xu et al., 2007). A previous study showed that oral administration of the strains from *Parabacteroides* could utilize polysaccharides, induce expression of interleukin-10, and helps maintain intestinal integrity, thus improve gut barrier function in mice (Wu et al., 2019). Enrichment of potential probiotics such as *Akkermansia* and *Parabacteroides* in WF mice suggests that the FMT from different donors could shape the bacteria composition, and intervention of dietary fiber could amplify this effect, thus contributed to the improvement of gut health. Nevertheless, we acknowledge that the antibiotic treatment could influence the effects of both FMT and dietary intervention in some of the results observed, especially the lower alpha diversity in FMT mice, although minor effects of antibiotic pretreatment in FMT were reported (Freitag et al., 2019). Although highly efficacious for the FMT in the treatment of dysfunction in several diseases were observed (Nowak et al., 2019; Chinna Meyyappan et al., 2020), 10–20% of recipients fail to achieve cure after a single FMT (Fischer et al., 2016; Lynch et al., 2019), our study demonstrated the diet intervention might contribute to the efficiency of FMT.

## Data Availability Statement

The datasets presented in this study can be found in online repositories. The names of the repository/repositories and accession number(s) can be found in the article/Supplementary Material.

## Ethics Statement

The animal study was reviewed and approved by the Animal Protection and Use Committee of Zhejiang University.

## Author Contributions

HW designed the experiments. JC, YZ, YM, and ZD performed the experiments. HW, YZ, JC, and JL analyzed the data. HW and YZ wrote and revised the main manuscript. All authors read and approved the final manuscript.

## Conflict of Interest

The authors declare that the research was conducted in the absence of any commercial or financial relationships that could be construed as a potential conflict of interest.

## Publisher’s Note

All claims expressed in this article are solely those of the authors and do not necessarily represent those of their affiliated organizations, or those of the publisher, the editors and the reviewers. Any product that may be evaluated in this article, or claim that may be made by its manufacturer, is not guaranteed or endorsed by the publisher.

## Footnotes

1
http://drive5.com/uparse/

2
http://rdp.cme.msu.edu/

## Supplementary Material

Supplementary Figure 1 Alpha diversity of Shannon **(A)** and Chao 1 **(B)** index, beta diversity **(C)**, composition **(D)**, cladogram **(E)** and LDA score **(F)** of gut microbiota LEfSe analysis and relative abundance of **(G)** of microbiota in the feces of wild (W) and domestic (DLY) pigs.

Supplementary Table 1 The composition of the purified diets and chow diet used in study.

Supplementary Table 2 Statistics of gut bacterial sequencing data in mice transplanted domestic pig fecal bacteria and feed a low-fiber diet (D) or high-fiber diet (DF), transplanted wild boar fecal bacteria and feed low-fiber diet (W) or high-fiber diet (WF).

Supplementary Table 3 Random forest model constructed for classification the mice transplanted domestic pig (D) and wild boar (W) fecal bacteria, the mean decrease accuracy of each bacteria genus in the model.

Supplementary Table 4 Random forest model constructed for classification the mice treated with low-fiber and high-fiber diet, the mean decrease accuracy of each bacteria genus in the model.
