# Supplementary figures and images for: Effect of Fiber and Fecal Microbiota Transplantation Donor on Recipient Mice Gut Microbiota
Source: Front Microbiol. 2021 Oct 13;12:757372. doi: 10.3389/fmicb.2021.757372 (PMC8548821; doi:10.3389/fmicb.2021.757372)

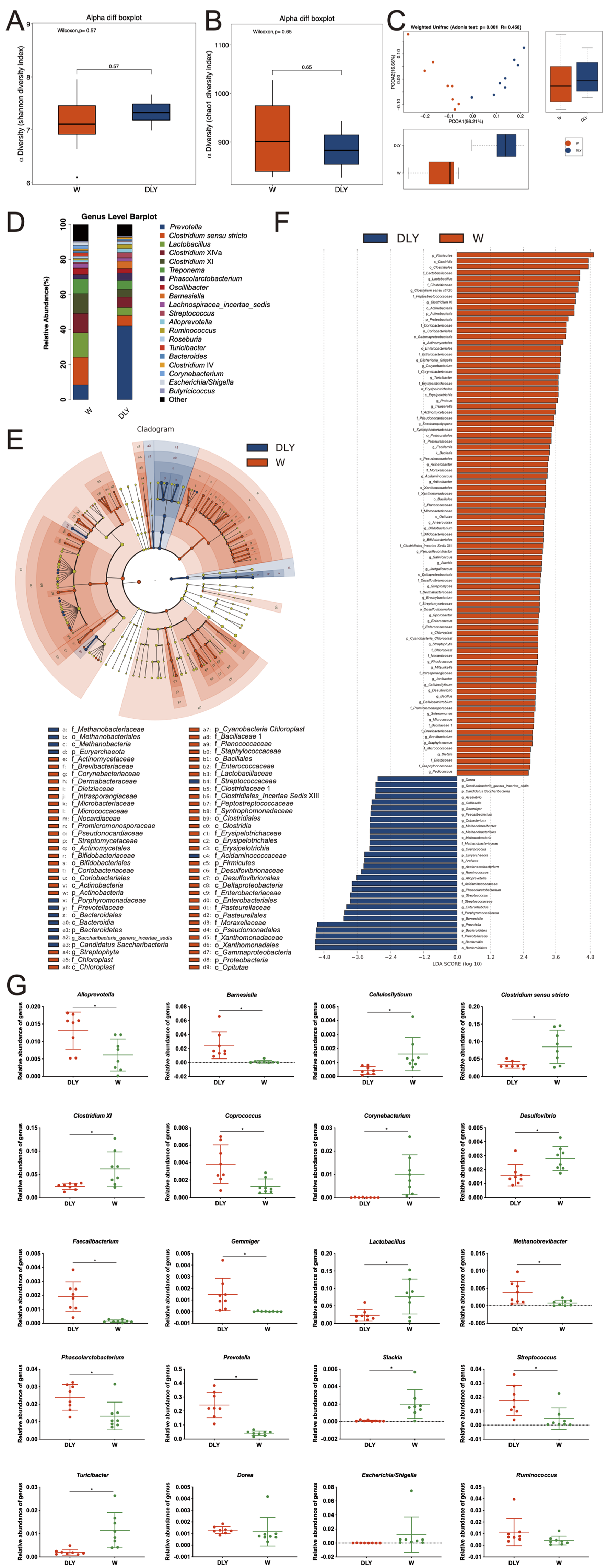

Supplement: Supplementary Figure 1 — Alpha diversity of Shannon (A) and Chao 1 (B) index, beta diversity (C), composition (D), cladogram (E) and LDA score (F) of gut microbiota LEfSe analysis and relative abundance of (G) of microbiota in the feces of wild (W) and domestic (DLY) pigs. [file Image_1.TIFF]
